# Current role of boron neutron capture therapy in head and neck cancer: insights from 4 years of clinical experience

**DOI:** 10.1016/j.ctro.2026.101316

**Published:** 2026-10-07

**Authors:** Satoshi Takeno, Yuki Yoshino, Teruhito Aihara, Takaki Inui, Tsuyoshi Jinnin, Ichita Kinoshita, Kazuma Kobata, Mari Mukai, Naonori Hu, Akinori Sasaki, Mai Nojiri, Yasukazu Kanai, Tetsuya Terada, Shin-Ichi Haginomori, Keiji Nihei, Koji Ono

**Affiliations:** a Department of Radiation Oncology, Osaka Medical and Pharmaceutical University, 2-7 Daigakumachi, Takatsuki, Osaka 569-8686, Japan; b Kansai BNCT Medical Center, Osaka Medical and Pharmaceutical University, 2-7 Daigakumachi, Takatsuki, Osaka 569-8686, Japan; c Department of Radiology, Kyoto Prefectural University of Medicine, 465 Kajii-cho, Kawaramachi Hirokoji, Kamigyo-ku, Kyoto 602-8566, Japan; d Department of Otorhinolaryngology - Head and Neck Surgery, Osaka Medical and Pharmaceutical University, 2-7 Daigakumachi, Takatsuki, Osaka 569-8686, Japan; e Department of Radiation Oncology, National Cancer Center Hospital East, 6-5-1 Kashiwanoha, Kashiwa, Chiba 277-8577, Japan; f BNCT Joint Clinical Institute, Osaka Medical and Pharmaceutical University, 2-7 Daigakumachi, Takatsuki, Osaka 569-8686, Japan; g Institute for Integrated Radiation and Nuclear Science, Kyoto University, 2-Asashiro-Nishi, Kumatori-cho, Sennan-gun, Osaka 590-0494, Japan

**Keywords:** Boron neutron capture therapy, Head and neck cancer, *Re*-irradiation

## Abstract

Purpose: Boron neutron capture therapy (BNCT) was approved in Japan in June 2020 as a curative option for unresectable, locally advanced, or recurrent head and neck cancers. Relevantly, its efficacy and safety in clinical practice should be regularly monitored, particularly regarding its optimal positioning. Therefore, to advance its clinical application, we assessed clinical data on BNCT for head and neck cancers.

Methods: We retrospectively analyzed 285 patients who received initial BNCT between June 2020 and May 2024 with available ≥1 month follow-up data. The primary endpoint was local control (LC); secondary endpoints included progression-free survival (PFS), overall survival (OS), treatment response, and grade ≥ 3 adverse events.

Results: The 2-year LC, PFS, and OS rates were 41.5%, 26.4%, and 59.2%, respectively. The complete response (CR), partial response (PR), and objective response (CR + PR) rates were 47.0%, 33.3%, and 80.4%, respectively. The cumulative incidence of grade ≥ 3 late adverse events was 14.0% at 2 years, with grade 5 adverse events occurring in 2.1% of patients. No significant differences were observed in LC or PFS among histological type subgroups; however, OS was significantly worse in patients with squamous cell carcinoma (SqCC) than in patients with non-SqCC. Regarding TNM stage at irradiation, radiation therapy at an early disease stage was significantly associated with improved LC.

Conclusions: BNCT should be administered during the early disease stages when no other effective treatment options are available. Further studies should determine the factors contributing to treatment efficacy and safety, thereby optimizing multimodal treatment strategies involving BNCT.

## Highlights

• We assessed clinical data on boron neutron capture therapy for head and neck cancer.
• It is effective at an earlier disease stage when curative treatment are unavailable.
• Its integration with other treatment modalities is essential.
• Factors contributing to its efficacy and safety should be further studied.

## Introduction

According to the GLOBOCAN 2022 estimates, head and neck cancers account for 4.7% of all tumors and are the seventh most common cancer type [1]. Standard curative treatments for these cancers currently include surgery and radiation therapy. Nevertheless, more than 50% of patients with locoregionally advanced disease experience recurrence within 3 years after treatment [2]. When surgery is not feasible and radiation therapy cannot be repeated, treatment becomes challenging. In such cases, drug therapy is typically administered. Although immune checkpoint inhibitor (ICI)–based therapies have demonstrated clinically meaningful activity in recurrent or metastatic HNSCC, objective response rates remain approximately 13–36% with currently available ICI-based regimens [2], [3]. Photon-based re-irradiation may be considered after multidisciplinary evaluation in carefully selected patients with recurrent head and neck cancer without distant metastases. Patient selection should take into account performance status, pre-existing organ dysfunction, tumor burden, the interval since previous radiotherapy, resection status, and cumulative dose constraints for critical organs [4], [5]. Clinically relevant acute toxicities of re-irradiation include mucositis, dermatitis, dysphagia, and aspiration. Late toxicities include persistent dysphagia, soft-tissue necrosis, fistula formation, osteoradionecrosis, cranial neuropathy, and, rarely, fatal carotid hemorrhage. Given these risks, re-irradiation should be performed in carefully selected patients at experienced institutions [6]. The inability to use surgery and radiation therapy makes curative treatment difficult in this setting. These highlight the urgent need for novel therapeutic approaches.

Boron neutron capture therapy (BNCT) is a novel treatment option for such patients [7]. In BNCT, a boron-containing drug enriched with boron-10 (^10^B) is administered and preferentially accumulates in tumor cells [8]. Subsequent neutron irradiation causes the ^10^B nuclei within these cells to capture neutrons, triggering the boron neutron capture reaction and producing alpha particles and recoiling lithium nuclei. These particles have an extremely short range of <10 μm and a high linear energy transfer, which enables the selective destruction of cells that have taken up the boron-containing drug. In June 2020, Japan became the first country to cover BNCT under the national health insurance system for the treatment of locally advanced or recurrent unresectable head and neck cancers [9], [10], [11]. Currently, several studies that aim to broaden the indications for BNCT are being conducted [12], [13], [14]. Additionally, more projects are being planned worldwide, raising expectations for the future widespread adoption of BNCT [15], [16], [17].

BNCT remains a novel treatment and warrants regular monitoring of its efficacy and safety in clinical practice. Accumulating evidence based on real-world clinical data is essential, particularly regarding its optimal positioning in standard head and neck cancer treatment protocols. Therefore, in this study, we aimed to collect and examine clinical data on BNCT for head and neck cancers to gain insights into its further advancement.

## Materials and methods

### Study design

In this retrospective study, we included patients with head and neck cancer who received BNCT under Japan's national health insurance system at Kansai BNCT Medical Center at Osaka Medical and Pharmaceutical University between June 2020 and May 2024. BNCT was performed following a previously reported protocol [11]. The analysis focused on patients with a minimum of 1 month of follow-up. The data cutoff date was December 2025. The referring physician determined the post-BNCT treatment plan. Post-BNCT follow-ups were conducted at our institution whenever possible. For patients who lived far away and had difficulty visiting the outpatient department, follow-up was performed at the referral facility, and information was collected by regularly sending prognosis questionnaires. If a patient had undergone BNCT twice, only the first treatment was included in the analysis.

This study was conducted in accordance with the content approved by the Ethics Committee of our institute (Study Number 2021-172). All patients who agreed to participate in the study provided written informed consent after receiving adequate information. Patients treated up to January 2022 were also included in the post-marketing surveillance conducted by Stella Pharma Corporation [18]. However, this study was conducted independently and Stella Pharma Corporation was not involved in designing the study, analyzing the data, interpreting the results or preparing the manuscript.

### Patient selection

In this study, we analyzed patients who met the eligibility criteria and received BNCT as an insurance-covered treatment. In Japan, BNCT is covered by the national insurance system for “unresectable locally advanced or recurrent head and neck cancers,” specifically in cases without malignant lesions outside the target area. Cases were deemed eligible for curative treatment if pre-planning based on diagnostic images indicated that the curative tumor-dose criterion described Appendix A/B could be achieved. However, cases with a high risk of carotid artery rupture or central nervous system invasion were excluded [19]. Effective standard treatments, such as surgery or radiation therapy, were prioritized when feasible.

### BNCT preparation and irradiation

For patients deemed eligible for treatment, a computed tomography (CT) simulation was performed based on the irradiation position determined in the pre-plan, and an irradiation plan was created according to Appendix B. In all cases, borofalan, a boronophenylalanine-based drug [20], was administered as the boron agent, followed by irradiation using the NeuCure BNCT System (Sumitomo Heavy Industries) [21].

Administration of the boron agent began at 200 mg/kg/h, starting 2 h before irradiation, and continued at a maintenance rate of 100 mg/kg/h from the start of irradiation. In BNCT, the absorbed dose from the boron neutron capture reaction is proportional to the tissue boron concentration and thermal neutron fluence. Therefore, the final neutron fluence required depends on the boron concentration. The blood boron concentration was measured immediately before irradiation using inductively coupled plasma atomic emission spectroscopy. However, despite administering boron at the maintenance rate, the blood boron concentration remained variable. Therefore, the blood boron concentration was measured again immediately after irradiation. The average of these two measurements was used as the actual boron concentration during irradiation. A post-plan was created using the actual irradiation parameters for the post-treatment dose assessment.

### Outcomes

The primary endpoint of this study was defined as local control (LC), defined as the time from the date of irradiation to the date of disease progression at the irradiated site. The secondary endpoints were progression-free survival (PFS), overall survival (OS), treatment response, and adverse events (grade 3 or higher). PFS was defined as the time from the date of irradiation to the date of disease progression (including that outside the irradiated field) or death. OS was defined as the time from the date of irradiation to the date of death from any cause. Treatment response was evaluated according to the Response Evaluation Criteria in Solid Tumors (RECIST) version 1.1, based on the best overall response: complete response (CR), partial response (PR), stable disease (SD), or progressive disease (PD) [22]. The minimum duration of SD was set at 3 months. If the disease progressed within 3 months without achieving a PR or better response, it was classified as PD. If subsequent therapy was initiated due to recurrence outside the irradiated field, the best overall response was evaluated up to the initiation of subsequent therapy. Data on adverse events were collected from medical records and follow-up questionnaires and evaluated according to the Common Terminology Criteria for Adverse Events (CTCAE) version 5.0 for events of grade 3 or higher.

### Data analysis

Data analysis was performed using Kaplan–Meier method, with LC as the primary endpoint and PFS and OS as secondary endpoints. Descriptive statistics were also used to analyze the treatment response and adverse events of grade 3 or higher. The cumulative incidence rate of late adverse events of grade 3 or higher was analyzed using Kaplan–Meier method. Late adverse events were defined as symptoms that appeared 3 months or more after BNCT, as well as earlier-appearing symptoms that persisted beyond 3 months.

To investigate dose–response relationships, we compared the distributions of the D_min_ and D_80%_ dose metrics across the four treatment response groups (CR, PR, SD, and PD). Each dose metric was first analyzed using the Kruskal–Wallis test. When a significant overall difference was identified, pairwise comparisons were performed using the Mann–Whitney *U* test with Holm correction for multiple comparisons.

As an exploratory analysis, we compared LC across subgroups defined by histological type, T and N categories in TNM classification (UICC/AJCC 8th edition) at irradiation, and treatment response using Kaplan–Meier method and log-rank test; PFS and OS were additionally evaluated only in the analysis by histological type. Moreover, for squamous cell carcinoma (SqCC) cases, we performed Cox proportional hazards regression analysis with TNM classification at irradiation, primary site, previous treatment history, and adjuvant treatment history as explanatory variables to investigate the factors related to the duration of LC. Among primary tumor sites, we used laryngeal cancer as the reference because it is one of the most common primary sites of head and neck cancer and represents the average treatment outcome in our preliminary review.

Statistical analysis was performed using Python version 3.10 (lifelines 0.27 library), and the statistical significance was set at 5% [23].

## Results

Between June 2020 and May 2024, 309 BNCT treatments were performed. Seven treatments were excluded because the patient's status at 1 month after the respective treatment could not be confirmed. Patients who died within 1 month after treatment were included. Of the remaining 302 treatments, 17 were second BNCT treatments and were excluded, leaving 285 initial BNCT treatments for the final analysis. These 285 cases (median patient age, 68 years; male-to-female ratio, 189:96) were analyzed in this study (Fig. S1). Treatments before BNCT included surgery (48.8%), radiation therapy (81.1%), chemotherapy (66.3%), and ICI therapy (28.3%). In this study, most patients had previously received a radiation therapy dose of 66–70 Gy. However, some patients had already undergone radiation therapy for other diseases; therefore, they did not receive radiation therapy for the disease being treated with BNCT. Fig. S2 summarizes the details of BNCT irradiation. The median blood boron concentrations before and after irradiation were 26.74 and 26.30 ppm, respectively. When the post-treatment doses were recalculated using the measured boron concentrations, they were estimated to range from 86.7% to 114.9% of the prescribed doses, indicating no systematic increase or decrease during irradiation. As pre-recurrence adjuvant therapy, chemotherapy and ICIs were administered to 3.2% and 6.0% of patients, respectively. The median observation period was 17.3 months (Table 1). Table 2 shows the breakdown of disease, TNM classification, and histological type.

**Table 1:** Patient characteristics.

Patient characteristics	
Median age (years) [range]	68 [24–93]
Males/Females	189 / 96

**Previous treatment**	
Surgery	139 (48.8%)
Radiation therapy	231 (81.1%)
Chemotherapy	189 (66.3%)
ICI	81 (28.3%)

**Adjuvant treatment after BNCT**	
Chemotherapy	9 (3.2%)
ICI	17 (6.0%)
No adjuvant treatment	260 (91.2%)
Median observation period (months) [range]	17.3 [0.7–63.4]

Abbreviations: ICI, immune checkpoint inhibitor; BNCT, boron neutron capture therapy.

**Table 2:** Disease details.

Cancer		Cases	TNM staging at irradiation									Pathology	
			T category					N category					
			T0	T1	T2	T3	T4	N0	N1	N2	N3	SqCC	Non-SqCC⁎
Oral cancer	Tongue	26	11	0	4	8	3	12	1	4	9	26	0
	Other	48	4	2	6	7	29	44	2	0	2	44	4
Nasopharyngeal cancer		15	5	4	6	0	0	9	3	0	3	14	1
Oropharyngeal cancer		46	12	13	12	2	7	29	4	3	10	44	2
Hypopharyngeal cancer		27	9	5	7	5	1	14	2	3	8	27	0
Laryngeal cancer		36	6	10	8	7	5	30	0	1	5	36	0
Nasal/paranasal cancer		29	1	2	3	6	17	27	2	0	0	21	8
Salivary gland cancer		16	2	2	1	5	6	11	1	0	4	1	15
Auditory cancer		29	2	0	2	9	16	25	1	1	2	28	1
Other cancer		13	7	1	2	1	2	6	0	1	6	8	5
Total		285	59	39	51	50	86	207	16	13	49	249	36

Abbreviations: SqCC, squamous cell carcinoma.

⁎ Non-SqCC includes acinar cell carcinoma (2 cases), adenocarcinoma (5 cases), adenoid cystic carcinoma (10 cases), carcinoma ex pleomorphic adenoma (1 case), carcinoma undifferentiated (1 case), malignant melanoma (2 cases), Merkel cell carcinoma (1 case), mucinous adenocarcinoma (1 case), mucoepidermoid carcinoma (4 cases), nuclear protein in testis (NUT)-associated carcinoma (1 case), olfactory neuroblastoma (2 cases), salivary duct carcinoma (3 cases), and spindle cell carcinoma (3 cases).

Among the 285 patients included in the analysis, the 1- and 2-year LC rates were 52.5% (95% confidence interval [CI]: 46.2–58.4%) and 41.5% (95% CI: 35.0–47.7%), respectively. The corresponding 1- and 2-year PFS rates were 39.4% (95% CI: 33.7–45.1%) and 26.4% (95% CI: 21.1–32.0%), respectively, and the corresponding OS rates were 78.6% (95% CI: 73.2–83.1%) and 59.2% (95% CI: 52.5–65.3%), respectively (Fig. 1). The median LC, PFS, and OS were 13.3, 8.8, and 33.5 months, respectively. In the overall cohort, the treatment response rates were 47.0% for CR, 33.3% for PR, 11.6% for SD, and 7.0% for PD. Three patients (1.1%) were not evaluable for treatment response. The objective response rate (ORR; CR + PR) was 80.4% (Table S1). The relationship between the dose indices (D_min_, D_80%_) and treatment efficacy is shown in Fig. S3; only a weak correlation was observed.

**Fig. 1:**
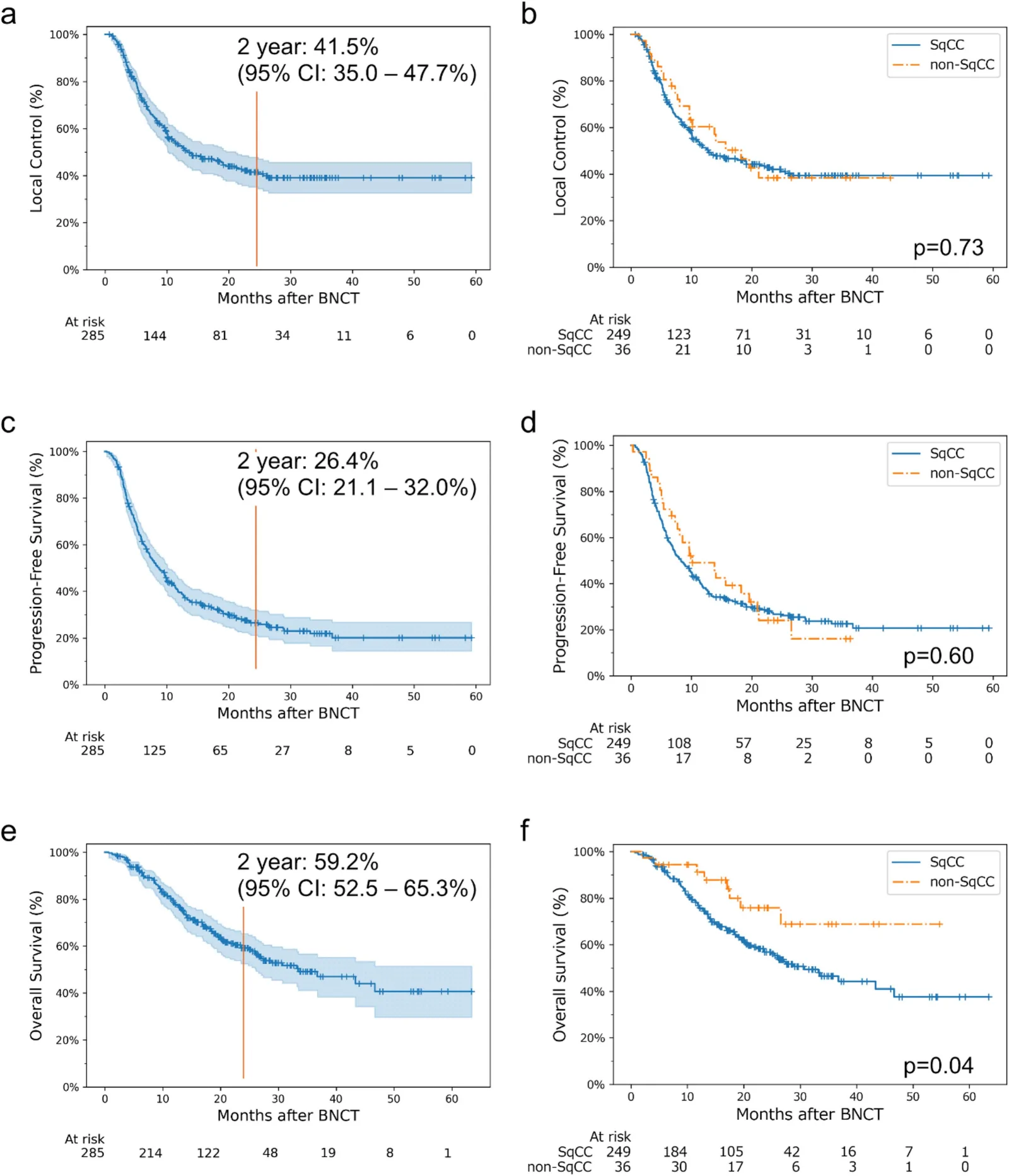
a and b, Kaplan–Meier curves for local control (LC); c and d, progression-free survival (PFS); and e and f, overall survival (OS). a, c, and e represent the combined results for all histological types, with 2-year LC, PFS, and OS rates of 41.5%, 26.4%, and 59.2%, respectively. b, d, and f were analyzed using the log-rank test for squamous cell carcinoma (SqCC) versus non-squamous cell carcinoma (non-SqCC), showing significantly worse OS for SqCC than for non-SqCC. Abbreviations: BNCT, boron neutron capture therapy; CI, confidence interval.

Analysis by histological type subgroups revealed no significant differences between the LC or PFS rates of the SqCC and non-SqCC groups. However, the non-SqCC group had a significantly better OS than the SqCC group when stratified and compared using log-rank test (Fig. 1). For SqCC, the 1- and 2-year rates were 51.3% (95% CI: 44.6–57.7%) and 42.0% (95% CI: 35.1–48.7%) for LC; 38.1% (95% CI: 32.0–44.2%) and 26.8% (95% CI: 21.1–32.7%) for PFS; and 76.8% (95% CI: 70.8–81.7%) and 56.9% (95% CI: 49.6–63.5%) for OS. The corresponding rates for non-SqCC were 60.4% (95% CI: 42.4–74.3%) and 38.3% (95% CI: 21.2–55.2%) for LC; 49.0% (95% CI: 31.8–64.2%) and 24.1% (95% CI: 10.6–40.4%) for PFS; and 91.2% (95% CI: 75.0–97.1%) and 75.8% (95% CI: 55.3–87.8%) for OS. In the SqCC group, the median LC, PFS, and OS were 12.8, 8.2, and 30.7 months, respectively. In the non-SqCC group, the median LC and PFS were 18.2 and 10.2 months, respectively, while the median OS was not reached. Among patients with SqCC, the corresponding CR, PR, SD, and PD rates were 48.6%, 33.7%, 9.6%, and 6.8%, respectively, with an ORR of 82.3%. Among patients with non-SqCC, the corresponding rates were 36.1%, 30.6%, 25.0%, and 8.3%, respectively, with an ORR of 66.7% (Table S1).

Fig. S4 shows the results of the analysis of the relationship between TNM classification at irradiation and LC in patients with SqCC using log-rank test. Notably, log-rank test showed significantly better results for the T category in the earlier stage and a better trend for the N category in the earlier stage. Fig. S5 shows the relationship between treatment response and LC in patients with SqCC. Patients who achieved CR had long-term LC, whereas those who achieved PR, SD, or PD experienced early recurrence.

Among the 285 patients, grade 3 or higher oral and pharyngeal mucositis occurred in 37 patients (13.0%) and eight patients (2.8%), respectively. Skin ulceration, primarily due to preexisting cutaneous tumor invasion at the time of irradiation, was observed in seven patients (2.5%). Fatal adverse events (grade 5) included pharyngeal hemorrhage in three patients (1.1%), esophageal perforation in one patient (0.4%), cranial nerve infection in one patient (0.4%), and sepsis in one patient (0.4%). In total, grade 5 adverse events were observed in 2.1% of cases. Table 3 presents all adverse events of grade 3 or higher. The cumulative incidence of such late adverse events at 2 years was 14.0% (Fig. 2). Details of Grade 5 cases are shown in Table S2.

**Table 3:** Adverse events (≥ grade 3).

	Grade 3	Grade 4	Grade 5	Total
**Acute adverse events**				
Acute kidney injury	5			5
Anemia	2			2
Anorexia	3			3
Mucositis oral	36	1		37
Pharyngeal mucositis	8			8

**Late adverse events**				
Aspiration	2			2
Central nervous system necrosis	1	1		2
Cranial nerve infection			1	1
Dysphagia	9			9
Esophageal perforation			1	1
Laryngeal edema⁎		2		2
Laryngeal fistula	1			1
Neck soft tissue necrosis	1	1		2
Osteonecrosis	1			1
Osteonecrosis of jaw	5			5
Pharyngeal fistula	2	1		3
Pharyngeal hemorrhage	1		3	4
Pharyngeal necrosis		1		1
Sepsis			1	1
Skin ulceration	5	2		7

(Total: 285 cases)

Asymptomatic increases in serum amylase that were clinically insignificant were excluded.

Other grade 3 acute adverse events were as follows: cheilitis; radiation dermatitis; diarrhea; hypertension; malaise; otitis media; soft tissue infection; and tumor pain. Other grade 3 late adverse events were as follows: hoarseness; retinopathy; vagus nerve disorder; and vascular disorder.

⁎ Patients with tracheostomies were excluded.

**Fig. 2:**
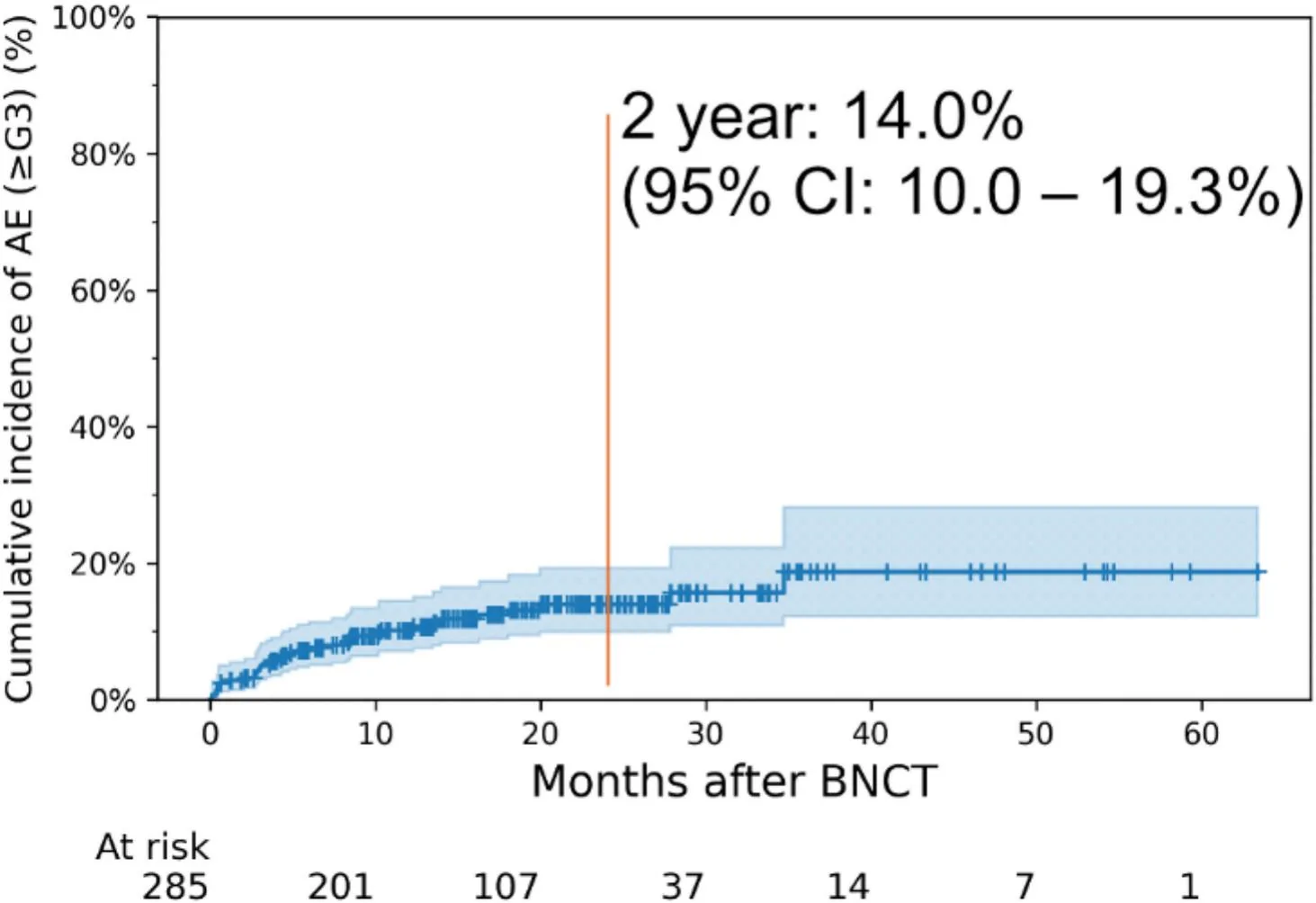
Cumulative incidence rate of late adverse events (AEs) of grade 3 or higher using Kaplan–Meier method. The result was 14.0% at 2 years.

Fig. 3 shows the results of Cox regression analysis for LC in SqCC cases. No significant violations of the proportional hazards assumption were detected in this analysis. These results confirmed that TNM classification was significantly associated with LC. Furthermore, the chemotherapy group showed an inferior trend of LC among previous treatment histories, whereas the ICI group showed a slightly better trend, although the difference was not significant. Regarding the primary site, the LC tended to be lower in nasopharyngeal cancer and higher in oral cavity cancer than in cancers of other primary sites.

**Fig. 3:**
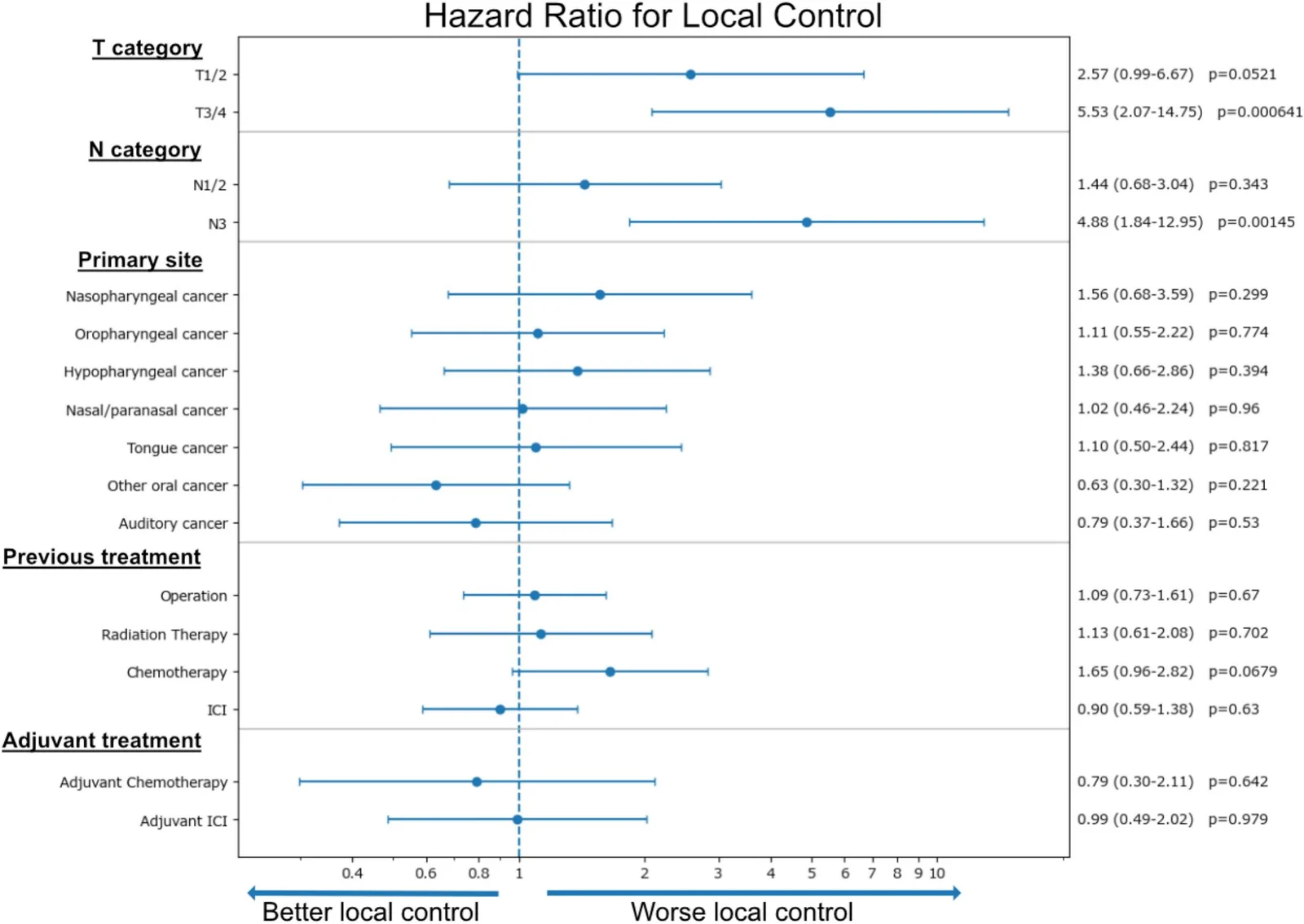
Results of Cox regression analysis for local control (LC) in squamous cell carcinoma cases. Advanced T and N categories in TNM classification at irradiation significantly decreased LC compared with that in other stages. Primary sites, such as oral cancer and auditory cancer sites, which are relatively superficial, showed a better trend of LC than did the other sites. In contrast, nasopharyngeal carcinoma, which is located relatively deep, showed a worse trend of LC than did the other sites. Here, laryngeal cancer was set as the reference for primary sites. Previous chemotherapy tended to decrease LC, whereas immune checkpoint inhibitors (ICIs) slightly increased it compared with that in the control.

## Discussion

For patients with advanced or recurrent head and neck cancer who are not eligible for curative surgery or conventional radiotherapy, systemic therapy is generally the standard treatment option. The ORR was 36.3% and the 2-year OS was 29.4% for SqCC patients who received platinum-based chemotherapy, including pembrolizumab, which is usually used as the first-line treatment, according to the results of KEYNOTE-048 [3]. The ORR decreases to 13.3% in patients treated with nivolumab for platinum-resistant disease, which is often used as second-line or later treatment (CheckMate 141) [2]. However, KEYNOTE-048 included patients with metastatic disease, whereas the present BNCT cohort was restricted to patients without malignant lesions outside the target region. In a post hoc analysis of the recurrent-only subgroup of KEYNOTE-040, which included patients without metastatic disease, the ORR was 22.6% with pembrolizumab and 16.7% with investigator's-choice standard therapy [24]. In our study, in which the patients met the above criteria, the ORR was 82.3% and 2-year OS was 56.9% in patients with SqCC, demonstrating a highly favorable outcome. This finding suggests that LC achieved with BNCT may contribute to improved survival. However, direct comparison with the outcomes reported in these studies is difficult because of substantial differences in disease extent and patient selection. Therefore, our findings should be interpreted with caution.

In cases of recurrence after curative radiotherapy, re-irradiation may be attempted. In recent years, treatment outcomes for re-irradiation have improved in clinical trials, especially owing to high-precision radiation therapy, such as intensity-modulated radiation therapy (IMRT). Lee et al. [25] conducted a meta-analysis of re-irradiation using IMRT and reported a 2-year LC of 51.9%, OS of 45.7%, and grade 5 adverse events in 3.1% of patients. Our results showed a slightly inferior LC but superior OS compared with this meta-analysis. However, differences in the histological composition of the study populations should also be considered when interpreting these findings and constitute a limitation of this comparison. Owing to differences in patient populations of the two studies, definitive conclusions cannot be drawn from this comparison. IMRT re-irradiation is not currently recognized as a standard treatment; therefore, careful consideration of its indications is required. In the meta-analysis conducted by Lee et al., the median annual case enrollment of each clinical trial was 8.1 patients (range: 2.3–24.2 patients per year). In contrast, our study involved routine, insurance-covered treatment rather than a limited number of cases and achieved an average case enrollment of 77.3 patients per year (maximum: 143 patients per year) at a single institution. These results suggest that BNCT, in routine clinical practice, achieves treatment outcomes similar to those of IMRT re-irradiation, which is typically reserved for a limited number of patients. We consider this a clinically significant finding.

The findings of this study suggest that early TNM classification at the time of irradiation leads to good treatment outcomes with BNCT. Skin and mucosal infiltration due to disease progression causes skin and mucosal ulcers and increases the risk of carotid artery rupture [19]. In this study, grade 3 or higher skin ulcers, pharyngeal fistula, esophageal perforation, and grade 5 carotid artery rupture were observed. These risks may be reduced by earlier treatment. Therefore, BNCT should be considered before disease progression makes curative treatments such as surgery or radiotherapy infeasible.

Oral cancers (excluding tongue cancer), which are typically more superficial, exhibited a favorable LC, whereas deep-seated tumors such as nasopharyngeal carcinoma exhibited a poorer LC. This site-dependent pattern is consistent with an inherent limitation of BNCT, in which the therapeutic neutron flux (and thus the delivered dose) attenuates with increasing tissue depth, potentially resulting in an insufficient tumor dose for deep-seated lesions. These findings highlight the need for improved neutron sources and irradiation techniques to enhance the therapeutic outcomes of BNCT. Potential strategies include the use of multiple neutron fields from different irradiation directions to compensate for reduced dose at greater depths, optimization of the neutron energy spectrum and neutron yield, and careful patient positioning to improve dose delivery to deep-seated tumors. In addition, achieving more efficient and homogeneous ^10^B uptake in cancer cells, potentially through the development of novel boron carriers, may further increase the tumor dose without requiring a corresponding increase in neutron fluence.

As shown in Fig. S3, the associations between conventional dose metrics and treatment outcomes were weak and of limited clinical relevance. Establishing a clinically meaningful dose–response relationship therefore remains an important challenge. One potential approach to addressing this issue is to incorporate the spatial distribution of boron into dose calculation and evaluation. Further studies are needed to develop ^18^F-FBPA-PET–based methods that account for spatial heterogeneity in boron distribution. Dose–toxicity relationships should also be investigated while accounting for cumulative normal-tissue doses from prior radiotherapy.

This study has some limitations inherent to its retrospective observational design. First, selection bias and confounding related to treatment assignment cannot be fully eliminated, and residual unmeasured confounders may remain despite statistical adjustment. Second, because outcomes were assessed based on medical record documentation, information bias and missing data are possible. Finally, as this was a single-center study, the generalizability of our findings may be limited. Despite these limitations, its results lay the groundwork for future studies. Prospective multicenter studies with standardized patient selection, boron administration, treatment planning, dosimetry, outcome assessment, and follow-up are needed to confirm reproducibility across institutions and neutron sources. Ultimately, randomized phase III trials comparing BNCT with appropriate contemporary re-irradiation approaches, with or without systemic therapy, should be considered.

In conclusion, our results suggest that BNCT should be administered during the early stages in patients with head and neck cancers when no other effective treatment options are available. As cancer treatment advances toward multimodal strategies, the integration of BNCT with other treatment modalities has become essential. Further studies, including prospective clinical trials, are needed to generate robust evidence and establish BNCT as a standard treatment.

## CRediT authorship contribution statement

**Satoshi Takeno:** Writing – original draft, Methodology, Investigation, Funding acquisition, Formal analysis, Data curation, Conceptualization. **Yuki Yoshino:** Investigation, Data curation, Conceptualization. **Teruhito Aihara:** Validation, Project administration, Investigation, Data curation, Conceptualization. **Takaki Inui:** Validation, Investigation, Data curation. **Tsuyoshi Jinnin:** Investigation, Data curation. **Ichita Kinoshita:** Investigation, Data curation. **Kazuma Kobata:** Investigation, Data curation. **Mari Mukai:** Investigation, Data curation. **Naonori Hu:** Investigation, Data curation. **Akinori Sasaki:** Investigation, Data curation. **Mai Nojiri:** Investigation, Data curation. **Yasukazu Kanai:** Investigation, Data curation. **Tetsuya Terada:** Validation, Investigation, Data curation. **Shin-Ichi Haginomori:** Validation, Investigation, Data curation, Conceptualization. **Keiji Nihei:** Writing – review & editing, Validation, Investigation, Data curation, Conceptualization. **Koji Ono:** Validation, Supervision, Investigation, Data curation, Conceptualization.

## Ethics declaration

Written informed consent to take part in the study and to publish the article has been obtained from all participants or their legal representatives. The privacy rights of participants have been observed.

This study was performed in compliance with relevant laws, regulatory frameworks and guidelines where the research took place.

This study was approved by the the Ethics Committee of Osaka Medical and Pharaceutical University.

(Approval No. 2021-172)

## Funding

This work was supported by 10.13039/501100001691JSPS KAKENHI Grant Number JP23K14878.

## Declaration of competing interest

The authors declare the following financial interests/personal relationships which may be considered as potential competing interests: Satoshi TAKENO reports financial support was provided by Japan Society for the Promotion of Science. Satoshi TAKENO reports travel was provided by Sumitomo Heavy Industries Ltd. If there are other authors, they declare that they have no known competing financial interests or personal relationships that could have appeared to influence the work reported in this paper.

## Data Availability

Research data are stored in an institutional repository and will be shared upon request to the corresponding author.

## Appendix A Calculation of biologically weighted dose in BNCT

For BNCT, the dose was evaluated as the biologically weighted dose (Gy-Eq), calculated by applying the compound biological effectiveness (CBE) or relative biological effectiveness (RBE) factors to the individual dose components and summing the weighted components [26]. Neutron and photon fluences were converted into the corresponding physical absorbed-dose components using energy-dependent KERMA and dose-conversion coefficients implemented in the treatment planning system. The physical absorbed dose was separated into four components: the boron dose (D_B_) resulting from the ^10^B(n,α)^7^Li reaction, the nitrogen dose (D_N_) resulting from the ^14^N(n,p)^14^C reaction, the hydrogen-recoil dose (D_H_) arising mainly from recoil protons generated by neutron interactions with hydrogen, and the photon dose (D_γ_), including primary and secondary photon contributions.

The biologically weighted dose (D_BW_) was calculated as:

D_BW_ = CBE × D_B_ + RBE_N_ × D_N_ + RBE_H_ × D_H_ + RBE_γ_ × D_γ_ [27].

The RBE factors for the nitrogen, hydrogen-recoil, and photon components were 2.9, 2.4, and 1.0, respectively. The CBE factors used for the tumor, pharyngeal mucosa, skin, and other normal tissues were 3.8, 4.9, 2.5, and 1.35, respectively [28], [29], [30].

For calculation of the boron-dose component, the ^10^B concentration in normal tissues was assumed to be equivalent to the measured blood ^10^B concentration, whereas the tumor ^10^B concentration was estimated using a tumor-to-blood concentration ratio of 2.5. Accordingly, the reported tumor and normal-tissue doses included all four physical dose components—boron, nitrogen, hydrogen-recoil, and photon doses—with the corresponding biological weighting factors applied.

## Appendix B Treatment planning

In BNCT treatment planning, the boron concentration in normal tissues was assumed to be the same as that in blood. Thus, the tumor boron concentration was calculated from the blood boron concentration using the tumor-to-blood boron concentration ratio (T/B ratio). According to the previous report, treatment effectiveness correlates with the minimum tumor dose [31]. Therefore, the T/B ratio was set to a minimum value of 2.5, as previously determined, to facilitate the calculation of the minimum tumor dose [32]. Under these parameters, the criterion for curative-intent tumor-dose coverage was defined as a gross tumor volume (GTV) D_80%_ of ≥20 Gy-Eq; that is, at least 80% of the GTV was required to receive a biologically weighted dose of ≥20 Gy-Eq. [11], [33], [34].

Although the T/B ratio and CBE factor are inherently tumor-specific, current clinical practice lacks methods to individualize these values for each patient. Consequently, these fixed values were used in the calculations, entailing tumor dose uncertainty. Therefore, treatment plans were developed based on the normal tissue tolerance, which is considered more reliable than the tumor dose, to maximize the tumor dose within the tolerance limits of normal tissues. The tolerance doses for normal tissues were set as follows: skin, 15 Gy-Eq; pharyngeal mucosa, 12 Gy-Eq; brain, 9 Gy-Eq; and eye, 5 Gy-Eq. [11]. In particular, the pharyngeal mucosa was delineated as a 2-mm-thick wall along the air cavity, with the contoured region limited to the portion within the irradiated field. For the pharyngeal mucosa, the 12 Gy-Eq tolerance was applied to D5%, rather than to the absolute maximum or mean dose.

The treatment plan was created using RayStation (RaySearch Laboratories AB, Stockholm, Sweden), and dose calculations were performed using the NeuCure Dose Engine (Sumitomo Heavy Industries, Tokyo, Japan) [35]. A 3-mm voxel size was used for dose calculation. Default material compositions implemented in RayStation based on ICRU/ICRP reference data were assigned to the tumor, skin, mucosa, brain, and eye. A CT-to-density calibration table was not used; instead, each ROI or structure was manually assigned the default physical density specified in RayStation. No patient-specific heterogeneity corrections were applied.
