# Supplementary material for: Current role of boron neutron capture therapy in head and neck cancer: insights from 4 years of clinical experience
Source: Clin Transl Radiat Oncol. 2026 Oct 7;62:101316. doi: 10.1016/j.ctro.2026.101316 (PMC13677289; doi:10.1016/j.ctro.2026.101316)
Supplement: Supplementary file 1 [file mmc1.docx]

**Supplementary material**

Table S1. Treatment responses

| **Treatment response** | **SqCC** | **non-SqCC** | **All** |
| --- | --- | --- | --- |
| CR | 121 (48.6%) | 13 (36.1%) | 134 (47.0%) |
| PR | 84 (33.7%) | 11 (30.6%) | 95 (33.3%) |
| SD | 24 (9.6%) | 9 (25.0%) | 33 (11.6%) |
| PD | 17 (6.8%) | 3 (8.3%) | 20 (7.0%) |
| NE | 3 (1.2%) | 0 (0.0%) | 3 (1.1%) |
|  |  |  |  |
| ORR | 82.3% | 66.74% | 80.4% |
| DCR | 92.0% | 91.7% | 91.9% |

Abbreviations: CR, complete response; PR, partial response; SD, stable disease; PD, progressive disease; NE, not evaluable; ORR, objective response rate (CR+PR); DCR, disease control rate (CR+PR+SD); SqCC, squamous cell carcinoma; non-SqCC, non-squamous cell carcinoma

Table S2. Details of Grade 5 cases

|  | Pharyngeal hemorrhage | | | Esophageal perforation | Cranial nerve infection | Sepsis |
| --- | --- | --- | --- | --- | --- | --- |
|  | Case 1 | Case 2 | Case 3 | Case 4 | Case 5 | Case 6 |
| Primary Site | Oropharynx | Hypopharynx | Oropharynx | Larynx | Auditory | Oral |
| Histology | SqCC | SqCC | SqCC | SqCC | SqCC | SqCC |
| TNM Classification | T1N0M0 | T2N1M0 | T2N0M0 | T0N3bM0 | T4N0M0 | T4bN0M0 |
| Invasion (Skin/mucosal/dura) | + | + | + | + | + | - |
| Proximity to the artery | + | + | + | + | - | - |
| Measured/Estimated boron concentration ratio | 89.3% | 101.3% | 100.3% | 97.9% | 94.4% | 97.2% |
| Dose metrix (Dmin-Dmax) (Gy-Eq) | 31.5-43.7 | 19.4-34.8 | 21.7-40.6 | 20.4-47.3 | 32.7-44.3 | 17.8-46.9 |
| Best overall response | CR | PR | CR | SD | PR | NE |
| Interval since prior radiotherapy (month) | 32.7 | 103.7 | 8.6 | 76.0 | 10.4 | 122.0 |
| Time to death (month) | 30.7 | 3.0 | 3.4 | 10.2 | 3.6 | 0.7 |

In Cases 1–4, the bleeding was considered to be associated with tumor shrinkage. In Case 5, the infection was considered to be attributable to intracranial tumor invasion. In Case 6, the relationship between sepsis and the tumor remained unclear.

Abbreviations: CR, complete response; PR, partial response; SD, stable disease; PD, progressive disease; NE, not evaluable

Figure S1. Flowchart depicting the selection of the study population. Between June 2020 and May 2024, 309 BNCT treatments were performed, comprising 27, 45, 94, and 143 treatments during the four successive 1-year periods from June to May, respectively. Seven treatments were excluded because the patient’s status at 1 month after the respective treatment could not be confirmed. Patients who died within 1 month after treatment were included. Of the remaining 302 treatments, 17 were second BNCT treatments and were excluded. Consequently, 285 initial BNCT treatments were included in the final analysis. Abbreviation: BNCT, boron neutron capture therapy.

Figure S2. The median blood boron concentrations before and after irradiation were similar at 26.74 and 26.30 ppm, respectively. The median measured-to-estimated boron concentration ratio, comparing the actual concentration with the pre-irradiation estimate used to determine the irradiation time, was 99.7%, suggesting no systematic over- or underestimation. The dose metrics for the target lesions are shown in this Figure.

Figure S3. The relationship between the dose indices (Dmin, D80%) and treatment efficacy. Individual dots represent individual patients, and the horizontal line within each violin indicates the median. Abbreviations: CR, complete response; PR, partial response; SD, stable disease; PD, progressive disease.

Figure S4. Kaplan–Meier curve for local control according to T and N categories in TNM classification at irradiation in patients with squamous cell carcinoma. log-rank test showed significantly better results for the T category in the earlier stage and a better trend for the N category than those for the other categories.

Figure S5. Relationship between treatment effects and local control in squamous cell carcinoma cases. CR is expected to lead to long-term control. However, non-CR (PR + SD + PD) cases are expected to experience early recurrence. Abbreviations: CR, complete response; PR, partial response; SD, stable disease; PD, progressive disease.
